# Verbal recognition of infants with cleft lip and palate with and without history of risk indicators for hearing loss

**DOI:** 10.1016/S1808-8694(15)30610-8

**Published:** 2015-10-18

**Authors:** Mariza Ribeiro Feniman, Bárbara Tavares Daniel, Luciana Paula Maximino De Vitto, Isabel Cristina Cavalcanti Lemos, José Roberto Pereira Lauris

**Affiliations:** 1 Associate Professor at the Speech and Hearing Therapy Department at Faculdade de Odontologia de Bauru, FOB-USP.; 2 Undergraduate degree in Speech and Hearing Therapy at Faculdade de Odontologia de Bauru da Universidade de São Paulo, FOB-USP, Speech and Hearing Therapist.; 3 PhD in Biology, Human Genetics. MD, Professor at the Department of Speech and Hearing Therapy at Faculdade de Odontologia de Bauru, FOB-USP.; 4 MSc in Speech and Hearing Therapy at the Department of Speech and Hearing Therapy at FOB-USP, Speech and Hearing Therapist.; 5 Associate Professor at the Department of Pediatric Dentistry, Orthodontics, and Collective Health at Faculdade de Odontologia de Bauru da Universidade de São Paulo, FOB/USP. Department of Speech and Hearing Therapy at Faculdade de Odontologia de Bauru da Universidade de São Paulo (FOB-USP).

**Keywords:** hearing, risk factors, cleft palate, ability, infant, recognition

## Abstract

The first two years of life are critical for the acquisition and development of hearing and speaking skills.

**Aim:**

This prospective study aims to verify the performance of infants with cleft lip and palate (CLP) with and without risk factors for hearing (RFH) in the verbal recognition test (VRT).

**Materials And Method:**

The parents of 100 infants (9 to 18 months of age) with CLP were interviewed to investigate the presence of RFH and to sort out the characteristics of the study groups. All infants underwent VRT.

**Results:**

Otologic diseases, lack of breastfeeding, parental smoking, upper airway insufficiency, stay in an incubator, and family history of hearing impairment were the most frequent RFH. Eighty-five infants had RFH, among which 40% had altered VRT results; fifteen did not have any RFH and 73% performed as expected for their age range in the VRT. There was no significant difference (p=0.326) between groups. Fifty-four infants had history of otitis media (OM), among which 31% had altered VRT results; forty-six had no history of OM and performed as expected for their age range in the VRT; Statistically significant difference (p=0.000) was found.

**Conclusion:**

Other risk factors for hearing aside CLP were found. Infants with and without history of RFH performed similarly in the VRT. The presence of otologic diseases significantly interfered with the VRT.

## INTRODUCTION

The first two years of life are considered to be a critical period for the acquisition and development of auditory and language skills.

Since the first weeks of life, a series of skills related to perception can be observed in infants. As is the case for other sensory organs, the vestibule is functional at birth. Infants are therefore able to locate sources of sound. However, starting at the third week of life, infants do not stay focused as they hear a sound^1^; they begin to be attracted to other things based on their own interests and experience. At the age of approximately one month, infants can identify a few peculiar traits of the human voice; they are able, for instance, to discern one voice from many, especially and logically that of their mothers.^2^

However, pre, intra, and post-natal disturbances may compromise a child's development. Damages inflicted upon the auditory sensory system will modify the way a child receives input, thus changing the nature of the intellectual and biopsychosocial experience he or she will have.^3^

The Joint Committee on Infant Hearing listed a series of risk indicators to identify the children at a higher risk of having hearing impairment.^4^ Among them are: family history of hearing loss, congenital infection, craniofacial anomalies, low weight, hyperbilirubinemia, ototoxic medication, bacterial meningitis, low Apgar score, being in assisted ventilation for at least 5 days, presence of syndromes associated with congenital hearing loss, prematurity, intracranial bleeding, and recurring or persistent secretory otitis media for at least three months, among others.

Longitudinal follow-up of infants at risk for developmental disorders is fundamental, as the first year of life represents a significant transition in the evolution of human beings, a time when the most important changes and evolutionary leaps take place.^5^

Considering therefore that the first year of life is critical in hearing development, that familiar voices are among the stimuli that best promote reliable responses from infants, and that congenital malformation is an important risk indicator for hearing and others, we decided to carry out a prospective study on the verbal recognition skills of infants with cleft lift and palate, with and without risk factors for hearing.

This study aims to verify the performance of infants with cleft lip and palate, with and without history of risk factors for hearing, tested for verbal recognition.

## MATERIALS AND METHOD

This study was approved by the Ethics Research Committee of our institution under permit 319/2004-UEPCEP. One hundred infants with ages ranging between nine and eighteen months were randomly enrolled in this prospective study. All infants included in this study had congenital cleft lip and palate and had undergone at least one lip or palate repair surgery. Nine infants had only their upper lips involved, sixty-eight had their upper lips and entire palates (soft and hard) involved, and twenty-three had only their palates (soft and hard) involved. This study was done in 2004 and 2005.

The parents of all infants were interviewed6 to check for hearing risk factors and split the infants into groups.

All parents read an Informative Letter and signed a Free Informed Consent Term.

All infants were examined for verbal recognition as proposed by Azevedo^7^. In this test, verbal commands are uttered in a natural, non-amplified fashion by the mother, as she stays 50 centimeters away in a lateral plane from the infant's ear, without giving visual cues, in a silent room.

The following verbal cues were used:

- Level 1: ‘Waive goodbye!’, ‘Throw me a kiss!’, ‘Clap your hands!’ - for 9-12 month old infants.
- Level 2: ‘Where's mommy?’, ‘Where's your pacifier?’, ‘Where's your shoe?’ - for 12-15 month old infants.
- Level 3: ‘Where's your hair?’, ‘Where's your hand?’, ‘Where are your feet?’- for 15-18 month old infants.

All responses were observed by an examiner and sorted as normal or altered. Normal response means the infant responded to the verbal cue expected for his or her age group, while altered means unresponsiveness.

Two groups were formed as a result of the interviews with the parents:

Group A: infants with risk factors for hearing impairment.
Group B: infants without risk factors for hearing impairment.

Statistical analysis was carried out using the chi-square test. A five percent threshold was adopted to refute the null hypothesis (Ho).

## RESULTS

**Chart 1:**
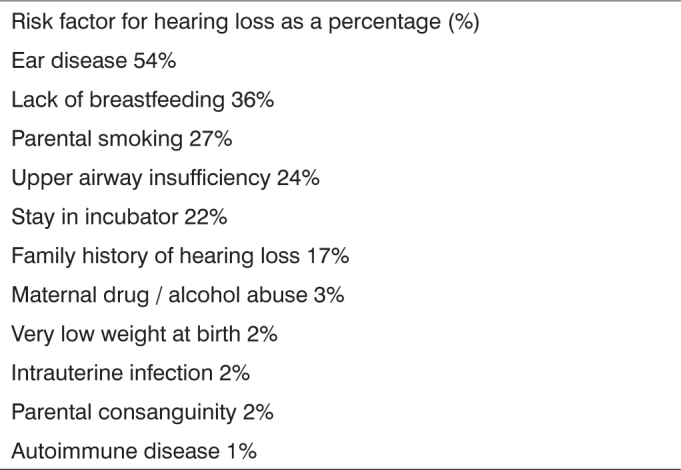
Distribution of risk factor for hearing loss.

After interviewing the parents of 100 infants, we found that 85 of the children had at least one risk factor for hearing impairment (Group A). The remaining fifteen were not reported for any hearing impairment risk factor (Group B). Chart 1 shows the distribution of such indicators.

**Chart 2:**
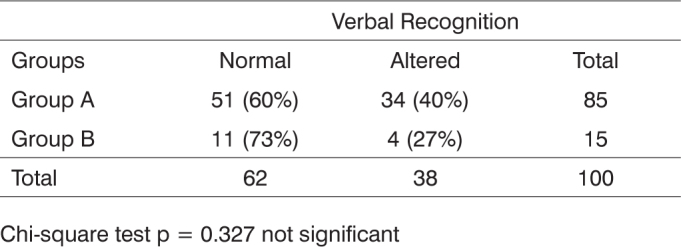
Distribution of responses to verbal cues per group.

Chart 2 presents the distribution, in absolute and relative terms, of the responses to the verbal recognition test done with the groups.

Chi-square test p = 0.327 not significant

**Chart 3:**
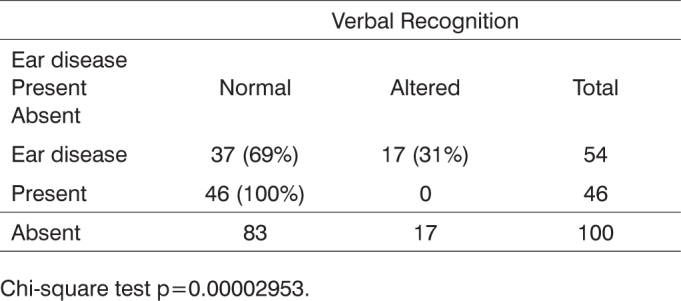
Distribution of responses to verbal cues per hearing impairment risk factor and ear diseases.

Statistical analysis pointed presence of ear disease (e.g.: otitis media) as the only risk factor with significant impact on the results of verbal recognition testing when compared to the other reported risk factors (Chart 3).

Chi-square test p=0.00002953.

## DISCUSSION

The Joint Committee on Infant Hearing (2000) stresses, among others, the presence of craniofacial malformations such as cleft lip and palate as a risk factor for hearing impairment. This study emphasized other risk factors found in the infants with cleft lip and palate (Chart 1).^4^

Otitis media is the most commonly diagnosed pediatric condition, affecting an estimated 70% of all children.8 Craniofacial malformations, specifically cleft lip and palate, establish a direct connection between the nasal and oropharyngeal cavities and the opening of the Eustachian tube. Therefore, Eustachian tube disorders affecting this population also present themselves as a risk factor for the development of secretory otitis media.^9^ The association between cleft lip and palate and disease is well documented, being secretory otitis media an almost universal finding in this congenital malformation^10^, ^11^, possibly explaining the significant percentage of ear diseases – otitis media being one of them – found in the studied population, as one of the most frequently observed risk factors for hearing impairment.

Evidences show that breastfeeding, especially when offered at least until the infant's sixth month of life, reduces the chance of otitis media, as the development of the face muscles and introduction of immunoglobulins act as protection mechanisms.^12^ Breastfeeding is a relevant factor in preventing or mitigating the occurrence of otitis media^13^, ^14^, ^15^. It also improves the infant's quality of life16 and acts as a means to transfer antibodies from the mother to the child, whose immune system is not sufficiently developed to tackle the pathogens present in the environment.^1^ As far as the studied population is concerned, lack of breastfeeding was found as a second indicator for risk of hearing impairment.

Exposure to tobacco smoke may lead to increased risk of respiratory disease (upper airway infections) and secretory otitis media among infants. Epidemiological findings and isolated studies point to increased prevalence and higher incidence of otitis media among children exposed to tobacco smoke. As the number of cases of respiratory infection in parents and infants increases, the more likely the episodes of otitis media become.^18^, ^19^ Second hand smoking leads to goblet cell hyperplasia accompanied by mucosal hypersecretion, and reduced mucociliary transport^20^, altering the nonspecific immune system and possibly producing a state of hypersensitivity.^21^ Smoking parents and upper airway infection were found in the population analyzed in this study and identified as risk factors for hearing impairment.

Exposure of newborns to intense noise in incubators7 and presence of diseases or conditions that require incubator stays longer than 48 hours4 are important risk factors for hearing loss that were also found in the group of infants enrolled in this study.

Epidemiological data suggests that congenital hearing loss occurs in one of every one thousand live births, with half of the cases having genetic origins.^22^ In this study, 17% of the sampled population reported a family history of hearing loss.

Maternal drug and alcohol abuse, low weight at birth, intrauterine infection, consanguine parents, and immune disease were some of the risk factors identified in this study, however with a prevalence rate below 3%.

Alcoholic drinks ingested by pregnant women go through the placenta barrier and expose the fetus to the same concentrations of alcohol found in the mother's bloodstream. Thus, as metabolism and elimination of substances is slower in the fetus, fetal exposure is dramatically increased.^23^ Sensorineural hearing loss in patients with fetal alcoholic syndrome was made evident by Church and Gerkin, and Church, Eldis, Blakley and Bawele.^24^, ^25^ In our study this risk factor had a prevalence rate of only 3%.

Individuals with cleft lip and/or palate are more likely to have very low weight at birth, however without establishing a direct relationship with prematurity^26^, and have more difficulty discriminating syllables and a consequent deficit in the central processing of speech sounds.^27^

Intrauterine infections including toxoplasmosis, measles, cytomegalovirus, herpes, and syphilis, have been pointed as relevant risk factor for hearing impairment and accounted for various degrees and patterns of hearing loss.^4^, ^28^, ^29^, ^30^, ^31^ In this study, these infections were acquired by some of the mothers during pregnancy. Immune disease was the least frequently found risk factor.

Significant associations between consanguine parents and cleft lip and palate have been described in the literature^32^, as well as between hearing loss and consanguinity.^33^, ^34^, ^35^, ^36^

Although Group A presents more altered results in the verbal recognition test, Group B had more responses within the level expected for the infants’ ages as proposed by Azevedo (1991)7 (Chart 2). As no statistically significant difference was found as the groups were compared, we may infer that the present risk factors for hearing loss did not impact the results infants had in the verbal recognition test. We could not find publications in the literature correlating risk factors for hearing loss and the test used in this study.

When statistically analyzing the impact of each risk factor on the verbal recognition test results, ear disease – otitis media, in the case – was the only risk factor bearing significant impact on the results of the verbal recognition test when compared to all other present factors.

History of ear disease as a risk factor for hearing impairment in the infants with cleft lip and palate analyzed in this study was the indicator that most impacted the performance of infants in the verbal recognition test. The deleterious impact on speech and language development introduced by the presence of otitis media has been mentioned in a number of studies.^37^, ^38^

## CONCLUSIONS

This study allowed the identification of other risk factors for hearing loss aside from congenital malformation. The performance of infants with and without history of risk factors for hearing loss was not different in the verbal recognition test. Presence of ear disease had statistically significant impact on verbal recognition test results.
